# Determination of Cobalt Spin-Diffusion Length in Co/Cu Multilayered Heterojunction Nanocylinders Based on Valet–Fert Model

**DOI:** 10.3390/nano11010218

**Published:** 2021-01-15

**Authors:** Saeko Mizoguchi, Masamitsu Hayashida, Takeshi Ohgai

**Affiliations:** 1 Graduate School of Engineering, Nagasaki University, Bunkyo-machi 1–14, Nagasaki 852-8521, Japan; bb52119647@ms.nagasaki-u.ac.jp; 2 Faculty of Engineering, Nagasaki University, Bunkyo-machi 1–14, Nagasaki 852-8521, Japan; hayashida@nagasaki-u.ac.jp

**Keywords:** anodization, nanochannel, electrodeposition, nanocylinder, cobalt, copper, heterojunction, multilayer, magnetoresistance, spin-diffusion length

## Abstract

Anodized aluminum oxide (AAO) nanochannels of diameter, *D*, of ~50 nm and length, *L*, of ~60 µm (*L*/*D*: approx. 1200 in the aspect ratio), were synthesized and applied as an electrode for the electrochemical growth of Co/Cu multilayered heterojunction nanocylinders. We synthesized numerous Co/Cu multilayered nanocylinders by applying a rectangular pulsed potential deposition method. The Co layer thickness, *t*_Co_, ranged from ~8 to 27 nm, and it strongly depended on the pulsed-potential condition for Co layers, *E*_Co_. The Cu layer thickness, *t*_Cu_, was kept at less than 4 nm regardless of *E*_Co_. We applied an electrochemical in situ contact technique to connect a Co/Cu multilayered nanocylinder with a sputter-deposited Au thin layer. Current perpendicular-to-plane giant magnetoresistance (CPP-GMR) effect reached up to ~23% in a Co/Cu multilayered nanocylinder with ~4760 Co/Cu bilayers (*t*_Cu_: 4 nm and *t*_Co_: 8.6 nm). With a decrease in *t*_Co_, (Δ*R*/*R*_p_)^−1^ was linearly reduced based on the Valet–Fert equation under the condition of *t*_F_ > *l*_F_^sf^ and *t*_N_ < *l*_N_^sf^. The cobalt spin-diffusion length, *l*_Co_^sf^, was estimated to be ~12.5 nm.

## 1. Introduction

Fert et al. and Grünberg et al. discovered the current-in-plane giant magnetoresistance (CIP-GMR) effect that the electric current passes through the in-plane direction of Fe/Cr multilayered thin films [1,2]. Schwarzacher et al. demonstrated the CIP-GMR effect by using the electrodeposited Co-Ni/Cu multilayered thin films [3]. After that, several research works have been reported that the electrodeposited ferromagnetic multilayered thin films exhibited the CIP-GMR effect [4,5,6,7]. However, considering an industrial application to a magnetic readout head in a hard disk drive (HDD), there are some issues concerning the quality of multilayered structure of an electrodeposited CIP-GMR device because it has a quite larger interface area (~10^−6^ m^2^) rather than the square of average crystal size (~10^−16^ m^2^).

On the contrary, a nanocylinder-based GMR sensor can realize an ideal sharp interface because the interface area (~10^−16^ m^2^) is a similar order to the square of average crystal size (~10^−16^ m^2^). These multilayered heterojunction nanocylinders with a large aspect ratio have a potential application to a magnetic readout head in a HDD, a magnetoresistive random access memory (MRAM) and high-sensitive metal-based magnetic field sensor with a small temperature coefficient (alternative to a Hall sensor), and so on. Piraux et al. and Blondel et al. demonstrated the current perpendicular-to-plane giant magnetoresistance (CPP-GMR) effect by using the Co/Cu multilayered nanocylinders which were electrodeposited into ion-track-etched polycarbonate membranes [8,9]. After that, several research works have been reported that the CPP-GMR effect was observed in the ferromagnetic multilayered nanocylinders which were electrodeposited into anodized aluminum oxide (AAO) templates [10,11,12,13,14,15,16,17]. Evans et al. reported that the Co-Ni/Cu multilayered nanocylinders, which were electrodeposited into commercially available AAO membranes (~300 nm in diameter, *D* and ~60 µm in length, *L*), exhibited a CPP-GMR effect of ~55% at room temperature [10]. They revealed that the Co-Ni alloy layer thickness, *t*_Co_ of about 5 nm and Cu layer thickness, *t*_Cu_ of about 2 nm were optimum values to exhibit a large CPP-GMR effect. Tang et al. also reported that the electrodeposited Co/Cu multilayered nanocylinders in commercial AAO templates showed a CPP-GMR effect of ~13.5% at room temperature [12]. They found that *t*_Co_ of ~8 nm and *t*_Cu_ of ~10 nm were optimum values to show a large CPP-GMR effect. Shakya et al. reported that the FeCoNi/Cu multilayered nanocylinders in commercial AAO templates showed a CPP-GMR effect of ~15% at room temperature [14]. Zhang et al. also reported that Ni-Fe/Cu/Co/Cu multilayered nanocylinders, which were electrodeposited into home-made AAO templates (*D* = 120 nm), exhibited a GMR effect of ~45% at room temperature [15]. Han et al. reported that the Co/Cu multilayered nanocylinders in home-made AAO templates (*D* = 50 nm) showed a CPP-GMR effect of ~13% at room temperature [16]. They revealed that *t*_Co_ of ~50 nm and *t*_Cu_ of ~5 nm were optimum values to demonstrate a large CPP-GMR effect. On the contrary, Xi et al. reported that the Co/Cu multilayered nanocylinders in home-made AAO templates (*D* = 80 nm) showed a small magnetoresistance effect of ~0.16% at room temperature [17]. The above research works have been conducted using AAO templates with an aspect ratio less than 250. It is estimated that the spin-valve response in the axial direction is improved by decreasing the nanocylinder diameter due to enhancing the magnetic shape anisotropy. Recently, we have demonstrated that Co/Cu multilayered nanocylinders, which were electrodeposited into a home-made AAO template (*D* = 75 nm and *L* = 70 µm), exhibited a CPP-GMR effect of ~23.5% at room temperature [18]. Hence, in the present study, to improve the CPP-GMR performance in the axial direction, we created Co/Cu multilayered nanocylinders electrodeposited into nanochannels with the diameter of ~50 nm (the aspect ratio is more than 1000). The spin-diffusion length in the cobalt layers was then determined based on the Valet–Fert equation.

## 2. Materials and Methods

A commercially available aluminum rod was mechanically and anodically polished in the cross-section (10 mm in diameter) to give a specular surface. During the anodic polishing process, bath voltage was maintained at 50 V for 120 s in an ethyl alcohol solution with 25 vol.% perchloric acid (HClO_4_) (FUJIFILM Wako Pure Chemical Corpo., Osaka, Japan). Afterward, to make an AAO nanochannel film, the polished cross-section was anodically oxidized in an electrolytic bath (0.3 mol/L oxalic acid) using a power supply (Bipolar DC Power Supply, BP4610, NF Corp., Yokohama, Japan). The nanochannel structure of an AAO film is strongly affected by anodization parameters [19,20]. In this study, the anodization voltage was kept at 50 V for 12 h. The AAO film was separated from an aluminum surface in an ethyl alcohol solution containing 50 vol.% perchloric acid (HClO_4_). During this separation process, the bath voltage was maintained at 55 V for 3 s. The separated films were employed as nanochannel templates for the electrodeposition of nanocylinders. To cover the nanochannels, a thick gold layer (250 nm) was formed on a surface of an AAO film using a DC magnetron sputter-deposition system (Auto Fine Coater, JFC-1600, JEOL Ltd., Tokyo, Japan). The thick gold layer works as a cathode in the nanochannels. A porous, thin gold layer (60 nm) was also formed on the other side surface of the AAO films without covering the nanochannels. The porous, thin gold layer functions as a floating electrode to make in situ contact with nanocylinders during electrodeposition. A pure gold wire was applied as a counter electrode, while an Ag/AgCl electrode was used as a reference electrode. An aqueous electrolytic solution was prepared using 0.5 mol/L cobalt (II) amido-sulfate (Co (SO_3_NH_2_)_2_ 4H_2_O) (Mitsuwa Chemicals Co. Ltd., Osaka, Japan), 0.005 mol/L copper (II) sulfate (CuSO_4_ 5H_2_O) (FUJIFILM Wako Pure Chemical Corpo., Osaka, Japan), 0.4 mol/L boric acid (H_3_BO_3_) (FUJIFILM Wako Pure Chemical Corpo., Osaka, Japan). The bath temperature was maintained at 40 °C, and the pH was adjusted to 4.0. To optimize the cathode potential for electrodeposition of Cu and Co layers, the linear sweep voltammetry technique was employed using an automatic polarization system (Electrochemical Measurement System, HZ-7000, Hokuto Denko Corp., Tokyo, Japan). Co/Cu multilayered nanocylinders with Cu layers (from 1.2 to 3.8 nm) and Co layers (from 7.8 to 26.8 nm) were grown into AAO nanochannels with an ultra-large aspect ratio of ~1200 using a rectangular pulsed-potential deposition process.

The bilayer thickness of Cu and Co was estimated from the AAO nanochannel length divided by the filling time. Each layer thickness of Co and Cu was determined from the bilayer thickness and the molar fraction using an energy-dispersive X-ray spectroscopy (EDX, EDX-800HS, Shimadzu Corp., Kyoto, Japan) and a field emission scanning electron microscopy with an energy-dispersive X-ray spectroscopy (FE-SEM-EDS, JSM-7500FA, JEOL Ltd., Tokyo, Japan). The constituent phases of the electrodeposited Co/Cu nanocylinders were investigated using an X-ray diffractometer (XRD, MiniFlex 600-DX, Rigaku Corp., Tokyo, Japan). After the electrodeposition, the nanocylinders were recovered from the AAO template by dissolving them in a sodium hydroxide aqueous solution (5 mol/L). The obtained nanocylinders were observed using a transmission electron microscope (TEM, JEM-2010-UHR, JEOL Ltd., Tokyo, Japan). Using the Co/Cu nanocylinders embedded in an AAO membrane, magnetization and magnetoresistance performance were evaluated using a vibrating-sample-magnetometer (VSM, TM-VSM1014-CRO, Tamakawa Co. Ltd., Sendai, Japan) and a source meter (DC voltage current source monitor, ADCMT6242, ADC Corp., Saitama, Japan). The magnetic field in-plane and perpendicular to the AAO film plane was applied while increasing the field up to 10 kOe. The perpendicular magnetic field corresponds with the axial direction of nanocylinders. The GMR value, *G*_MR_, can be defined by the following Equation (1).

(1) $$G_{MR}=\frac{R^{AP}-R^{P}}{R^{P}}$$

Here, *R*^P^ is the resistance with a maximum magnetic field of 10 kOe*,* and *R*^AP^ is the resistance without a magnetic field.

## 3. Demagnetization Factor and Valet–Fert Model in Multilayered Heterojunction Nanocylinders

The demagnetized field, *H*_d_, can be expressed by the following Equation (2).

(2) $$H_{d}=\left(\frac{N_{d}}{\mu_{0}}\right)\times I$$

Here, *N*_d_ is a demagnetization factor, *µ*_0_ represents a magnetic permeability in a vacuum, and *I* stands for the magnetization strength. *N*_d_ can be expressed by Equation (3) as a function of aspect ratio, *k* = *L*/*D* (*L*: nanocylinder length, *D*: nanocylinder diameter).

(3) $$N_{d}=\frac{1}{k^{2}-1}\left\{\frac{k}{\sqrt{k^{2}}-1}\ln\left(k+\sqrt{k^{2}-1}\right)-1\right\}$$

If a nanocylinder has a diameter *D* of 50 nm and length *L* of 60 µm, the aspect ratio, *k* = *L*/*D*, is 1200. In this case, the demagnetization factor, *N*_d_, can be estimated to be 4.3 × 10^−6^, which is almost zero. The spin-valve response in the axial direction will be improved by reducing the demagnetizing field with increased magnetic shape anisotropy.

Based on the Valet–Fert theory, under the conditions of *t*_F_ > *l*_F_^sf^ and *t*_N_ < *l*_N_^sf^, the spin-valve type GMR value has an inverse proportional relationship with the ferromagnetic layer thickness, *t*_F_, as shown by the following Equations (4)–(6) [21,22,23].

(4) $$\frac{R^{P}}{R^{ap}-R^{p}}=\frac{\left(\frac{\rho_{F}^{*}}{\rho_{F}^{e}}-{\left(\beta^{e}\right)}^{2}\right)}{2p{\left(\beta^{e}\right)}^{2}l_{F}^{sf}}t_{F}$$

(5) $$\rho_{F}^{e}=\rho_{F}^{*}+\rho_{mix}$$

(6) $$\beta^{e}=\frac{\beta }{\left[1+\frac{\rho_{mix}}{\rho_{F}^{*}}\right]}$$

Here, *R*^p^ and *R*^ap^ are resistance with and without a magnetic field, respectively, while *t*_F_, and *l*_F_^sf^ are the thickness of ferromagnetic layers and spin-diffusion length, respectively. *ρ*_F_* and *ρ*_mix_ are the resistivity and spin mixing resistance of ferromagnetic layers, respectively. *β* is the asymmetric coefficient of bulk scattering spin, and *p* is the constant ranging from 0.33 to 0.49. Piraux et al. reported that *β*^e^, *ρ*_F_*, and *ρ*_F_^e^ were 0.31 ± 0.02, 25 µΩcm, and 29 µΩcm, respectively, in their study on Co/Cu multilayered nanocylinders (*D* = 90 nm), which were electrodeposited from a sulfuric acid solution at room temperature. The ferromagnetic metal spin-diffusion length, *l*_F_^sf^, can be obtained from the approximate expression slope using the experimental data of present study.

On the contrary, under the condition of *t*_F_ < *l*_F_^sf^ and *t*_N_ < *l*_N_^sf^, the GMR value has the following relationship with the non-magnetic layer thickness, *t*_N_, shown in Equation (7).

(7) $${\left(\frac{R^{ap}-R^{p}}{R^{ap}}\right)}^{-1/2}=\frac{\rho_{F}^{*}t_{F}+2r_{b}^{*}}{\beta \rho_{F}^{*}t_{F}+2\gamma r_{b}^{*}}+\frac{\rho_{N}^{*}t_{N}}{\beta \rho_{F}^{*}t_{F}+2\gamma r_{b}^{*}}$$

Here, *ρ*_N_* represents the non-magnetic layer resistivity. *r*_b_* represents interface resistance. In contrast, *γ* is the asymmetric coefficient of the interface spin. Consequently, Equations (4) and (7) can be simply expressed as the following Equations (8) and (9). Here, *a*, *b*, and *c* mean proportional constants.

(8) $$\frac{R^{p}}{R^{ap}-R^{P}}=c\times t_{F}$$

(9) $${\left(\frac{R^{ap}-R^{p}}{R^{ap}}\right)}^{-1/2}=a\times t_{N}+b$$

In this study, the thickness of ferromagnetic layer, *t*_F_, was varied to determine the spin-diffusion length in the ferromagnetic layer according to Equation (8).

## 4. Results and Discussion

### 4.1. Template Synthesis and Electrodeposition Process of Co/Cu Heterojunction Nanocylinders

**Figure 1:**
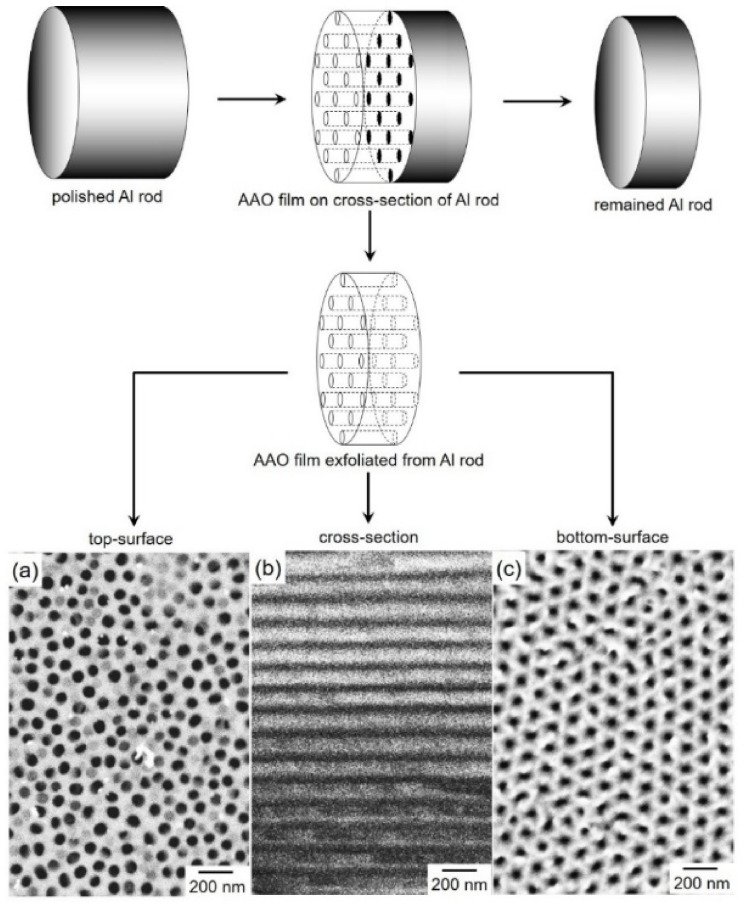
FE-SEM images of top-view (**a**), cross-section (**b**), and bottom-view (**c**) of an anodized aluminum oxide nanochannel template which was exfoliated from the cross-section of an aluminum rod.

Figure 1 shows the FE-SEM images of the top-side view (Figure 1a), the cross-sectional view (Figure 1b), and the bottom-side view (Figure 1c) of an AAO nanochannel film that separated from a cross-section of an aluminum rod. The separated AAO film had an ideal nanochannel structure with ~50 nm in diameter. The nanochannel length, which is identical to the AAO film thickness, was ~60 µm.

**Figure 2:**
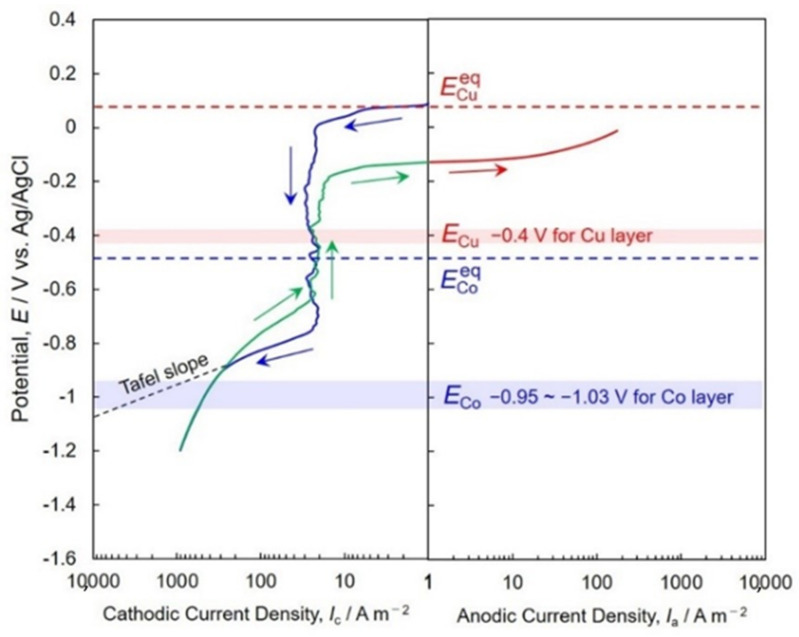
Cathodic (blue line) and anodic (green and red lines) scanned polarization curves (Tafel slope) for Cu and Co electrodeposition from an aqueous solution containing 0.5 M Co (SO_3_NH_2_)_2_·4H_2_O, 0.005 M CuSO_4_·5H_2_O and 0.4 M H_3_BO_3_.

Figure 2 shows the cathodic (blue line) and anodic (green and red lines) scanned polarization curves (Tafel slope) for Cu and Co electrodeposition from an aqueous solution containing Cu^2+^ and Co^2+^ ions. The Tafel plot was then employed to reveal the reduction behavior of Cu^2+^ ions by magnifying the relatively small current range. According to the Nernst equation, *E*_Cu_^eq^ for Cu/Cu^2+^ is estimated to be +0.07 V vs. Ag/AgCl, while *E*_Co_^eq^ for Co/Co^2+^ is also calculated to be −0.48 V vs. Ag/AgCl, as follow by Equation (10).

(10) $$E^{eq}=E^{0}+\frac{RT}{nF}\ln\left[M^{n+}\right]$$

Here, *E*^eq^ and *E*^0^ are the equilibrium potential and standard potential, respectively. *R*, *F*, *n,* and *T* are gas constant, Faraday constant, ionic valence, and absolute temperature, respectively. [*M^n^*^+^] is the activity of the metal ions. As shown in Figure 2 (cathodic scan: blue line), the cathode current density starts to rise at +0.07 V, which is close to *E*_Cu_^eq^. It is well known that the normal metal ions, such as Cu^2+^, Sn^2+^, Zn^2+^ ions are immediately reduced to the metallic state without substantial overvoltage in an acidic aqueous solution [24]. Hence, this cathode current rising results from Cu^2+^ ions’ reduction.

The cathode potential significantly polarizes to −0.80 V at the current density of around 23 A m^−2^. In the range of current density, Cu^2+^ ions seem to reach a diffusion limit. Moreover, an increase in the cathode current density can be observed at −0.80 V, which is quite less noble than *E*_Co_^eq^. It is well-known that Co^2+^ ions are reduced to a metallic state, accompanying a substantial overvoltage owing to the multi-step reduction process, which was reported by Bockris et al. [25]. Furthermore, in the potential region less noble than −1.2 V, the current density reached over 1000 A m^−2^_,_ and the cathode potential polarized significantly due to the diffusion limit of Co^2+^ ions [26]. On the contrary, in the anodic scan (green and red lines), the anodic current was observed at −0.13 V. This current seems to be caused by the dissolution of electrodeposited Co. For the pulsed potential deposition of Co/Cu multilayers, the suitable cathode potential for Cu layer, *E*_Cu_ should be less nobler than *E*_Cu_^eq^ (+0.07 V) and initial dissolution potential for Co (−0.13 V). Additionally, *E*_Cu_ should be nobler than *E*_Co_^eq^ (−0.48 V) to avoid Co contamination. Hence, in the present study, *E*_Cu_ was fixed to −0.4 V, while the suitable cathode potential for Co layer, *E*_Co_ should be less nobler than *E*_Co_^eq^ (−0.48 V) and initial deposition potential for Co (−0.80 V). To prevent Cu contamination, quite less nobler potential than −0.80 V is desirable for Co deposition. Moreover, *E*_Co_ should be nobler than the diffusion limit potential for Co^2+^ ions (−1.2 V). In this study, Co layer thickness should be controlled within the several tens of nanometer range to investigate the spin-diffusion length based on the Valet–Fert equation. Therefore, *E*_Co_ was determined to the range from −0.95 V~−1.03 V.

**Figure 3:**
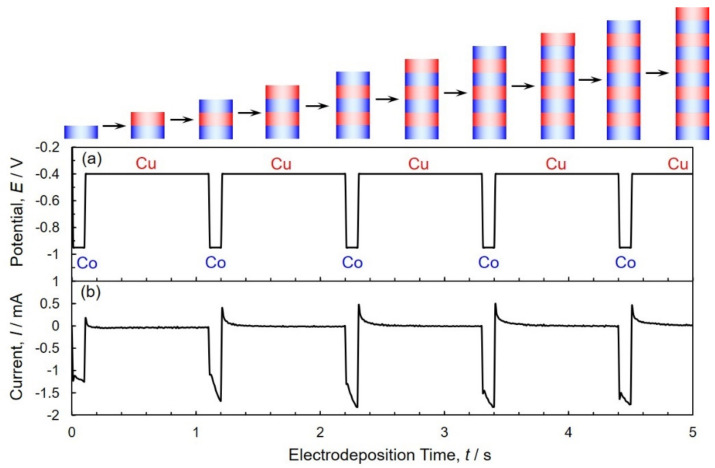
Time-dependence of applied potential (**a**) and observed current (**b**) at the beginning of pulsed-potential deposition time for growing Co/Cu multilayered nanocylinders. The cathode potential was alternatingly changed between −0.4 V (1.0 s) and −0.95 V (0.1 s).

**Figure 4:**
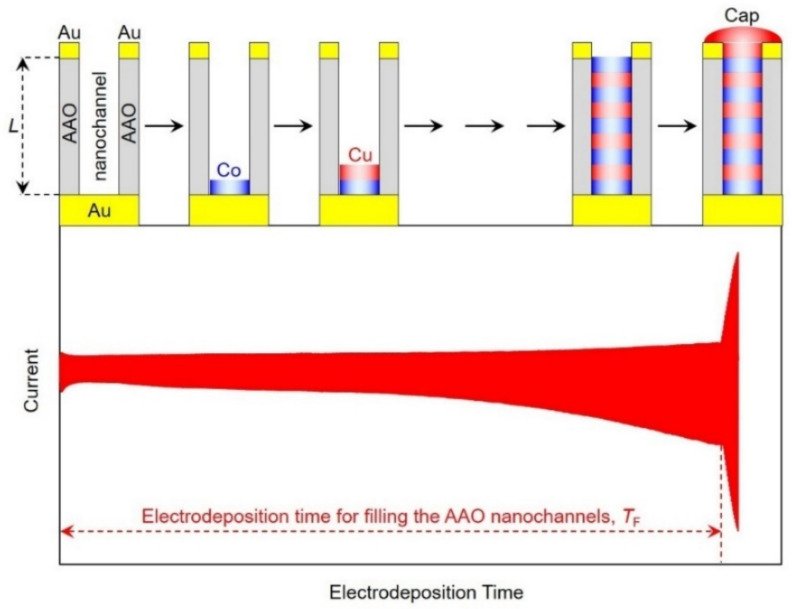
Schematic image for filling the AAO nanochannels with Co/Cu multilayered nanocylinders at the wide range of pulsed-potential deposition time.

As shown in Figure 3, we synthesized Co/Cu multilayered nanocylinders by switching the cathode potential from −0.4 V (for 1.0 s) to −0.95 V~−1.03 V (for 0.1 s) to adjust the thickness of each layer within several nanometer scale. When the nanocylinders reached the Au thin layer on an AAO template, the reduction current was suddenly enhanced due to the in-situ electric contact with the Au thin layer and formation of hemispheric metal caps as shown in Figure 4. The time for filling AAO nanochannels with Co/Cu multilayered nanocylinders, *T*_F_, was determined from the time-dependence of observed current at the wide range of pulsed-potential deposition time as shown in Figure 4.

The growth rate of Co/Cu multilayered nanocylinders, *R*_g_, can be estimated from dividing the AAO nanochannel’s length, *L*, by the filling time, *T*_F_. Furthermore, Co/Cu bilayer thickness, *t*_Co/Cu_, can be also estimated from the following Equation (11).

(11) $$t_{Co/Cu}=L\frac{T_{Co}+T_{Cu}}{T_{F}}$$

Here, *T*_Co_ and *T*_Cu_ are the pulse-deposition time for each Co and Cu layer, respectively. In the present study, *T*_Co_ and *T*_Cu_ correspond to 0.1 s and 1.0 s, respectively.

**Figure 5:**
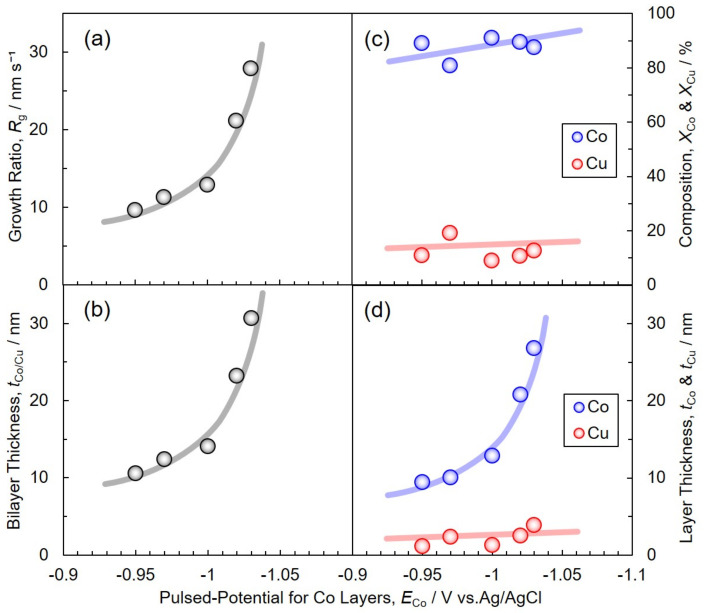
Effects of pulsed-potential for Co layers, *E*_Co_, on the growth rate of nanocylinders, *R*_g_ (**a**), Co/Cu bilayer thickness, *t*_Co/Cu_ (**b**), the average composition, *X*_Co_ and *X*_Cu_ (**c**), and the average layer thickness, *t*_Co_ and *t*_Cu_ (**d**). *T*_Co_, *E*_Cu_ and *T*_Cu_ were fixed to 0.1 s, −0.40 V and 1.0 s, respectively.

Figure 5a,b show the effect of *E*_Co_ (pulsed potential for Co layer deposition) on the nanocylinder growth rate, *R*_g_ and Co/Cu bilayer thickness, *t*_Co/Cu_, respectively. When *E*_Co_ was shifted to the less noble region, *R*_g_ and *t*_Co/Cu_ increased logarithmically up to 27.9 nm s^−1^ and 30.7 nm, respectively. Based on Tafel equation (*η* = *a* + *b*log*i*), the overpotential, *η*, is proportional to the logarithm of current, log*i*, when the charge transfer process controls the electrochemical reaction. It is well-known that the nanocylinder growth rate and bilayer thickness are a linear relationship with the electrodeposition current density based on Faraday’s laws of electrolysis. Hence, *R*_g_ and *t*_Co/Cu_ should be increased logarithmically with increasing the overpotential. The composition of Co, *X*_Co_ and that of Cu, *X*_Cu_ in each sample were also determined from EDX analysis (EDX-800HS, Shimadzu, Kyoto, Japan) as shown in Figure 5c. All over the potential range from −0.95 V to −1.03 V, the average *X*_Co_ and *X*_Cu_ were 87.58% and 12.42%, respectively. The compositions were also investigated by FE-SEM-EDS analysis (JSM-7500FA, JEOL, Tokyo, Japan). The average *X*_Co_ and *X*_Cu_ were also determined to 87.96% and 12.04%, respectively. If the Cu impurities in Co layers are negligible, each average layer thickness of Co and Cu, *t*_Co_ and *t*_Cu_, can be estimated from the following Equations (12) and (13), respectively.

(12) $$t_{Co}=t_{Co/Cu}\frac{X_{Co}}{100}$$

(13) $$t_{Cu}=t_{Co/Cu}\frac{X_{Cu}}{100}$$

The effect of *E*_Co_ on *t*_Co_ and *t*_Cu_ is shown in Figure 5d. The *t*_Cu_ was almost constant at less than 4 nm all over the potential range. On the other hand, *t*_Co_ became thicker as *E*_Co_ was shifted to a less noble region. According to the above results, it was revealed that *t*_Co_ can be controlled within the range from 8 to 27 nm by tuning *E*_Co_.

### 4.2. Structure of Co/Cu Heterojunction Nanocylinders

**Figure 6:**
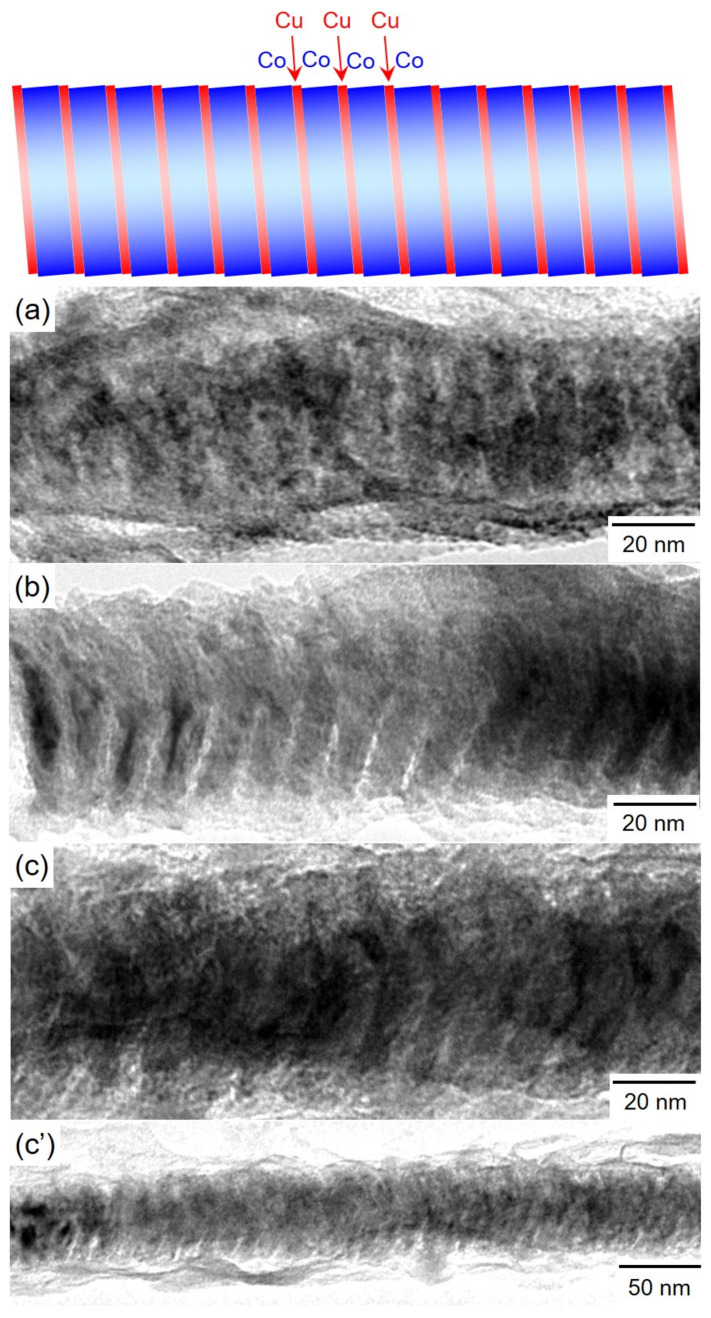
TEM images of Co/Cu multilayered nanocylinders that were separated from an anodized aluminum oxide nanochannel template. (**a**) *E*_Co_ = −0.95 V, (**b**) *E*_Co_ = −0.97 V, (**c**,**c’**) *E*_Co_ = −1.00 V. *T*_Co_, *E*_Cu_ and *T*_Cu_ were fixed to 0.1 s, −0.40 V and 1.0 s, respectively.

Figure 6 shows TEM bright-field images of Co/Cu multilayered nanocylinders. The samples were prepared by ranging the pulsed-potential for Co layer, *E*_Co_ as the following: Figure 6a *E*_Co_ = −0.95 V, Figure 6b *E*_Co_ = −0.97 V and Figure 6c,c’ *E*_Co_ = −1.00 V. While the other parameters: *T*_Co_, *E*_Cu_ and *T*_Cu_ were fixed to 0.1 s, −0.40 V and 1.0 s, respectively. The Co/Cu multilayered nanocylinders were separated from AAO templates. As shown in Figure 6, the diameter of Co/Cu multilayered nanocylinder is ~50 nm, which is almost identical to the diameter of AAO nanochannels as shown in Figure 1. The nanocylinder also has a multilayered heterojunction structure. The layer thickness of a dark thick layer is ~10 nm while that of a light thin layer is ~2 nm. The thick and thin layers correspond to the Co and Cu layers, respectively, considering the estimated layer thickness, as shown in Figure 5d.

**Figure 7:**
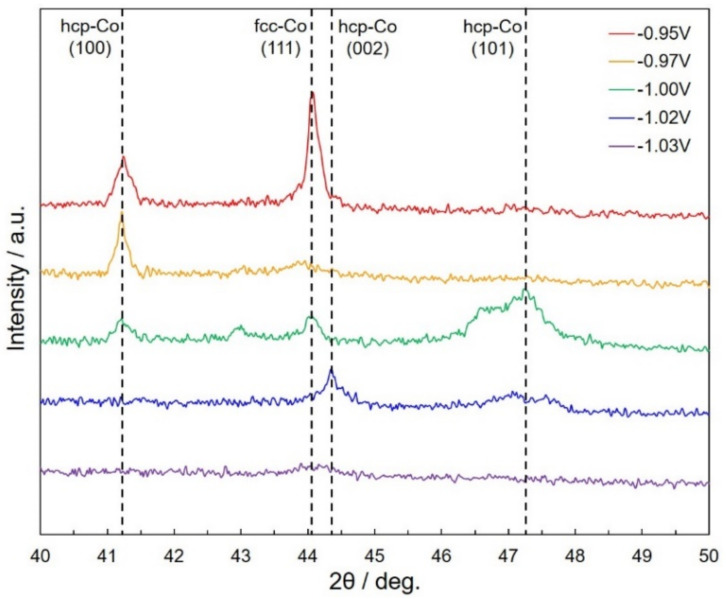
Effect of pulsed-potential for Co layer, *E*_Co_ on the X-ray diffractograms of Co/Cu multilayered nanocylinders. *E*_Co_ was set for −0.95 V, −0.97 V, −1.00 V, −1.02 V and −1.03 V. *T*_Co_, *E*_Cu_ and *T*_Cu_ were fixed to 0.1 s, −0.40 V and 1.0 s, respectively.

Figure 7 renders the effect of *E*_Co_ on the XRD profiles of Co/Cu multilayered nanocylinders. As shown in Figure 7, the observed peaks at 2*θ* = 41.25°, 44.1°, 44.4°, and 47.25° are derived from hcp-Co (100), fcc-Co (111), hcp-Co (002), and hcp-Co (101), respectively. The diffraction peak, which is derived from fcc-Co, is observed at 2*θ* = 44.1°. The presence of fcc-Co could be caused by the phase transformation from the hcp to the fcc structure because a part of the Co layer seems to contain Cu as the impurity element. Other researchers have also reported that the fcc-Co phase existed in the X-ray diffraction pattern on their Co/Cu multilayered films [27]. In contrast, the diffraction peak of fcc-Co disappeared when the pulsed potential for Co layer was set to a less nobler region. The peak disappearance results from an increase in the Co layer thickness, as shown in Figure 5d.

### 4.3. Magnetoresistance Properties of Co/Cu Multilayered Heterojunction Nanocylinders

**Figure 8:**
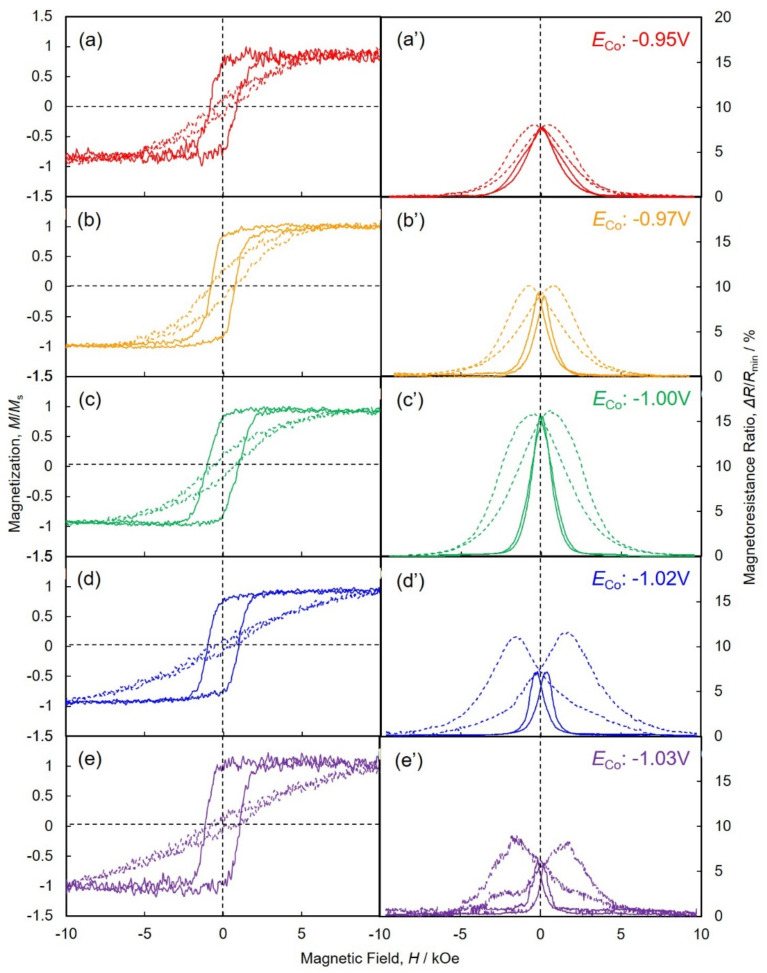
Effect of pulsed-potential for Co layers, *E*_Co_ on the magnetic and magnetoresistance hysteresis loops of AAO nanochannel films with Co/Cu multilayered nanocylinder arrays. *E*_Co_ was set for −0.95 V (**a**,**a’**), −0.97 V (**b**,**b’**), −1.00 V (**c**,**c’**), −1.02 V (**d**,**d’**) and −1.03 V (**e**,**e’**). *T*_Co_, *E*_Cu_ and *T*_Cu_ were fixed to 0.1 s, −0.40 V and 1.0 s, respectively. The magnetic field was applied to in-plane (dotted lines) and perpendicular (solid lines) directions to the multilayer interfaces.

The effect of *E*_Co_ on the magnetic and magnetoresistance hysteresis curves of Co/Cu multilayered nanocylinder arrays is shown in Figure 8. The hysteresis curves, which were obtained in the magnetic field perpendicular to the AAO film, are plotted in the solid lines, while the curves that obtained in-plane direction are plotted in the dotted lines. As shown in the dotted lines of Figure 8a–d, it is quite difficult to achieve the saturation magnetization with a magnetic field in-plane direction to the AAO film due to a substantial demagnetizing field, *H*_d_. The demagnetization factor, *N*_d_ with in-plane direction can be estimated to ~0.5. On the other hand, as shown by the solid lines, it is relatively easy to achieve the saturation magnetization with a perpendicular magnetic field to the AAO film plane. As shown in Equation (2), *H*_d_ will be minimal in a perpendicular direction, which corresponds to the axial direction of a nanocylinder. In this case, the external magnetic field will be effective and not reduced. Hence, the saturation magnetization can be realized by a small external magnetic field (~2 kOe) in the long axis direction of nanocylinders [28].

If the resistance of a multilayered structure can be expressed by the linear relationship with the composition, the resistance of a Co/Cu multilayered nanocylinder can be defined using the resistivities of a Co layer and a Cu layer as shown in Equation (14).

(14) $$R=\left(\rho_{Co}\frac{X_{Co}}{100}+\rho_{Cu}\frac{X_{Cu}}{100}\right)\frac{L}{S}$$

Here, *R* is the resistance of a Co/Cu nanocylinder. *ρ*_Co_ and *ρ*_Cu_ are the resistivity of a Co layer (64.2 Ω/nm) and a Cu layer (16.8 Ω/nm), respectively. *L* and *S* are the length (60 µm) and the cross-section area (~6360 nm^2^) of a nanocylinder, respectively. According to Equation (14), *R* will increase with increasing *X*_Co_ because *ρ*_Co_ is larger than *ρ*_Cu_. Based on our experimental results, the resistance of a Co/Cu multilayered nanocylinder, which was synthesized by an electrochemical in situ contact process, corresponded to the estimated value for the parallel contacts with only 1~3 nanocylinders regardless of the composition.

As shown by the dotted lines of Figure 8a–d, the magnetoresistance of Co/Cu multilayered nanocylinder arrays decreased like a Gaussian curve. The resistance reached the minimum in the range more than ~7 kOe as the magnetic field increased slowly in the in-plane (parallel) direction. On the other hand, the magnetoresistance ratio decreased quickly and reached zero at ~2 kOe with an increasing magnetic field in the perpendicular (axial) direction, as shown by the solid lines.

**Figure 9:**
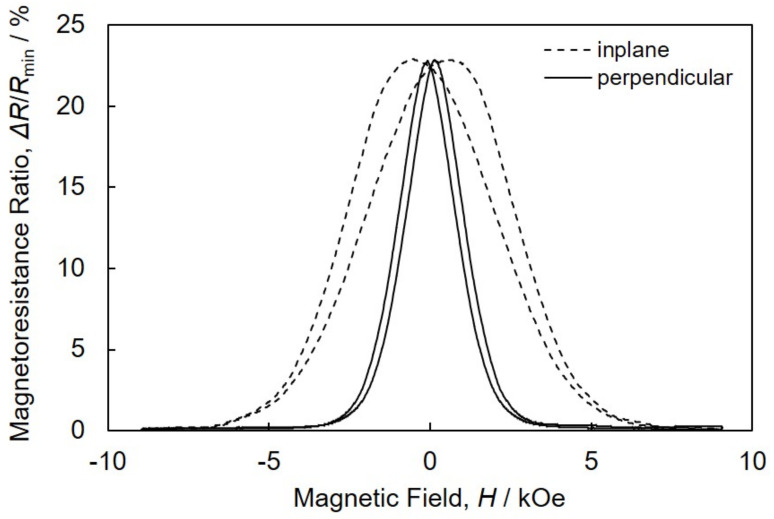
Magnetoresistance hysteresis loops of an AAO nanochannel film with Co/Cu multilayered nanocylinder arrays. The sample was synthesized using the pulse parameters of *E*_Co_ = −1.05 V, *T*_Co_ = 0.03 s, *E*_Cu_ = −0.4 V and *T*_Cu_ = 1.0 s. The magnetic field was applied to in-plane (dotted lines) and perpendicular (solid lines) directions to the multilayer interfaces.

The GMR value of Co/Cu multilayered nanocylinder arrays, which were electrodeposited at *E*_Co_ of −1.03 V, was ~9%, as shown in Figure 8e’. While, the GMR value of the nanocylinder arrays, which were electrodeposited at *E*_Co_ of −1.00 V, increased up to ~16%, as shown in Figure 8c’. It has been reported that the GMR value increases as the number of interfaces between ferromagnetic and non-magnetic layers increases [29]. As shown in Figure 5d, the Co layer thickness became thinner as the pulsed potential was shifted to a noble region. This decrease in the Co layer thickness increases the number of layer interfaces. Hence, this increase in GMR seems to be caused by decreases in the Co layer thickness. For further improving the CPP-GMR performance, the Co layer thickness, *t*_Co_ was decreased by shortening the pulse-deposition time for Co layer, *T*_Co_. To maintain the throwing power for the pulse-deposition, the pulsed-potential for Co layer, *E*_Co_ was kept to less nobler than −1.03 V. Figure 9 show the magnetoresistance hysteresis loops of an AAO nanochannel film with Co/Cu multilayered nanocylinder arrays. The nanocylinder arrays were electrodeposited using the pulse parameters of *E*_Co_ = −1.05 V, *T*_Co_ = 0.03 s, *E*_Cu_ = −0.4 V and *T*_Cu_ = 1.0 s. As shown in Figure 9, the CPP-GMR value reached up to ca. 23% in the Co/Cu multilayered nanocylinder with 8.6 nm in *t*_Co_ and 4 nm in *t*_Cu_.

Table 1 shows the summary of CPP-GMR performances (at room temperature) of electrodeposited multilayered nanocylinders that were reported by the other researchers. Most researchers have reported that the CPP-GMR value reached up to ca. 15~20% at room temperature in the *t*_Co_ range from ca. 5 to 20 nm and the *t*_Cu_ range from ca. 5 to 10 nm. Those values give good agreement with the value obtained in the present study.

**Figure 10:**
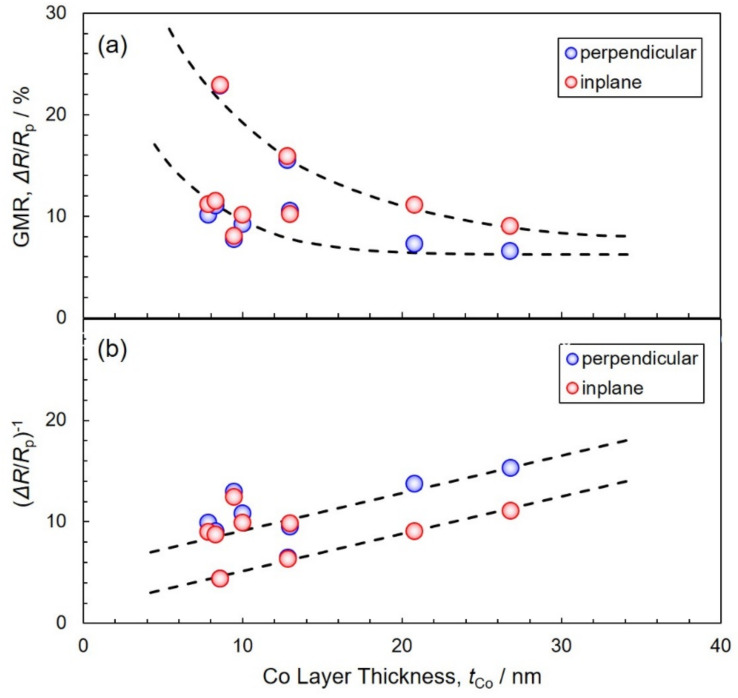
Effect of Co layer thickness on GMR (**a**) and (*ΔR*/*R*_p_)^−1^ (**b**) in electrodeposited Co/Cu multilayered nanocylinders.

Figure 10 shows the effect of Co layer thickness on the GMR value and (Δ*R*/*R*_p_)^−1^ of Co/Cu multilayered nanocylinders. As shown in Figure 10a, the GMR value increases with a decreasing Co layer thickness. In the Co layer thickness of 8.6 nm, the GMR value reached up to ~23%. As shown in Figure 10b, with a decrease in the thickness of the Co layer, (Δ*R*/*R*_p_)^−1^ decreases linearly [13]. This tendency corresponds well to Valet–Fert Equation (8). The spin-diffusion length of magnetic metal can also be estimated from the slope of approximate expression in Figure 10b. Consequently, the cobalt spin-diffusion length, *l*_Co_^sf^, was estimated to be ~12.5 nm. As the thickness of the Co layer, *t*_Co_, is from 8 to 27 nm, the condition of *t*_F_ > *l*_F_^sf^ in the Valet–Fert model seems to be satisfied by the results in the present study.

## 5. Conclusions

AAO nanochannel films (*D*: ~50 nm, *L*: ~60 µm) were fabricated using an anodization and exfoliation technique from a metallic aluminum rod. The Co/Cu multilayered nanocylinders were fabricated by alternating the cathode potentials for Cu and Co deposition to adjust the Co layer thickness within ~30 nm. From the TEM images of the Co/Cu multilayered nanocylinders, it was confirmed that the Co and Cu layers were alternately laminated, and the diameter of the nanocylinders was the same as the pore diameter of the AAO template. The multilayered nanocylinders with alternating Cu and Co layers contained both hcp and fcc phases of cobalt. The multilayered nanocylinders with alternating Cu and Co layers reached saturation magnetization with a small magnetic field (~2 kOe) in the axial direction of nanocylinders due to the substantial aspect ratio. As the Co layer thickness decreased, the GMR reached up to approx. 23%. When decreasing the Co layer thickness, (Δ*R*/*R*_p_)^−1^ linearly decreased according to the Valet–Fert equation; this can be explained under the condition of *t*_F_ > *l*_F_^sf^ and *t*_N_ < *l*_N_^sf^. The cobalt spin-diffusion length, *l*_Co_^sf^, was estimated to be ~12.5 nm by the slope of approximate expression.

## Figures and Tables

**Table 1:** Summary of CPP-GMR performance (at room temperature) of multilayered nanocylinders electrodeposited into AAO that were reported by the other researchers. The nanocylinders in Refs. [8,9] were electrodeposited into ion-track-etched polycarbonate membranes.

Authors	FM/NM	GMR/%	*D*/nm	*L*/μm	*L*/*D*	*t*_Co_/nm	*t*_Cu_/nm	Source Title	Year	Ref.
Piraux et al.	Co/Cu	15	40	10	250	10	10	*Appl. Phys. Lett.*	1994	[8]
Blondel et al.	Co/Cu	14	80	6	75	5	5	*Appl. Phys. Lett.*	1994	[9]
Evans et al.	CoNi/Cu	55	300	60	200	5	2	*Appl. Phys. Lett.*	2000	[10]
Ohgai et al.	Co/Cu	15	60	2	33	10	10	*J. Appl. Electrochem.*	2004	[11]
Tang et al.	Co/Cu	14	300	60	200	8	10	*J. Appl. Phys.*	2006	[12]
Tang et al.	CoNi/Cu	23	300	60	200	10	4	*Phys. Rev. B*	2007	[13]
Shakya et al.	FeCoNi/Cu	15	300	60	200	14	10	*J. Magn. Magn. Mater*	2012	[14]
Zhang et al.	FeNi/Cu/Co	45	120	2	17	25	15	*J. Mater. Sci. M. E.*	2015	[15]
Han et al.	Co/Cu	13	50	11	220	50	5	*Adv. Cond. Mat. Phys.*	2016	[16]
Xi et al.	Co/Cu	0.16	80	3	38	200	5	*Physica B*	2017	[17]
Kamimura et al.	Co/Cu	24	75	70	933	19	1.4	*Nanomaterials*	2020	[18]
